# The potential of Andaliman (*Zanthoxylum acanthopodium* DC) fruit as an ethanol extract for neuroprotection in aged model rat

**DOI:** 10.5455/javar.2023.j713

**Published:** 2023-12-31

**Authors:** Dwi R. Anggraini, Syafruddin Ilyas, Poppy A. Z. Hasibuan, Yetty Machrina, Tri Widyawati, Rusdiana Rusdiana, Fitriani Lumongga, Suryani E. Mustika

**Affiliations:** 1 Department of Anatomy, Faculty of Medicine, Universitas Sumatera Utara, Medan, Indonesia; 2 Department of Biology, Faculty of Mathematics and Natural Sciences, Universitas Sumatera Utara, Medan, Indonesia; 3 Department of Pharmacology, Faculty of Pharmacy, Universitas Sumatera Utara, Medan, Indonesia; 4 Department of Physiology, Faculty of Medicine, Universitas Sumatera Utara, Medan, Indonesia; 5 Department of Pharmacology and Therapeutic, Faculty of Medicine, Universitas Sumatera Utara, Medan, Indonesia; 6 Department of Biochemistry, Faculty of Medicine, Universitas Sumatera Utara, Medan, Indonesia; 7 Department of Anatomical Pathology, Faculty of Medicine, Universitas Islam Sumatera Utara, Medan, Indonesia

**Keywords:** Aging model rats, BDNF, spatial memory, VEGF, Zanthoxylum acanthopodium DC

## Abstract

**Objective:**

Dementia is a common aging-related neurodegenerative disease in the elderly worldwide. Alterations in neurogenesis and angiogenesis factors have been linked to cognitive impairment in neurological disorders. However, synthetic drugs to improve memory disorders have uncomfortable side effects. The purpose of this study is to explore the neuroprotective potential of the fruit ethanol extract of andaliman (*Zanthoxylum acanthopodium* DC) [Andaliman fruit ethanol extract (AEE)] on brain-derived neurotrophic factor (BDNF), vascular endothelial growth factor (VEGF), and spatial memory in rat models of aging.

**Materials and Methods:**

This study had an experimental design with AEE. The 4 groups were treated as follows: N (normal), M (served as positive control), P1 (AEE 150 mg/kg bw), and P2 (AEE 300 mg/kg BW) for 8 weeks. Aged model rats (M, P1, and P2) were obtained by inducing D-galactose (150 mg/kg bw). BDNF and VEGF expression were determined by RT-PCR, and spatial memory was assessed using the test of the Moris Water Maze (MWM). The Kruskal–Wallis and Mann–Whitney tests were used to assess the statistical analysis.

**Results:**

AEE had a tendency to increase BDNF in P2 compared to the normal group (1.98 versus 1). VEGF expression increased in P1 and P2 compared to the normal group (1.14 and 1.29 versus 1). AEE at a dose of 300 mg/kg bw significantly improved spatial memory (*p* = 0.026).

**Conclusion:**

For eight weeks, AEE at a dose of 300 mg/kg bw considerably increased the potential to enhance VEGF and BDNF expression as well as spatial memory.

## Introduction

D-galactose, as one of the chemical compounds induced by aging, has been used as an aging model in animal studies [1,2]. Overconsumption of D-galactose can activate three different metabolic pathways of D-galactose, such as the Leloir pathway, conversion of hydrogen peroxide, D-galacto-hexodiadose, and galactitol via galactose oxidase and reductase [3]. Accelerated aging may be explained by the buildup of these products in cells, which leads to oxidative stress and osmotic pressure. Several studies using experimental animals as aging models have demonstrated that D-galactose induction enhances oxidative stress and brain tissue apoptosis [4].

Brain-derived neurotrophic factor (BDNF), as a strong neuroprotective agent to prevent neurodegenerative processes, induces neurogenesis, and synaptic plasticity, and modulates the synaptic structure, which is important in learning, high-order thinking, mood, and affect regulation [5]. BDNF stimulation has been reported to have both increased growth and proliferation of cells in the hippocampus, which are important in memory formation and long-term potentiation (LTP) [6]. Expression of BDNF, which is active in the cortex, cerebri, hippocampus, and basal forebrain, has been shown to reduce brain aging [7]. Previous studies have shown that hippocampus volume decreases with decreasing plasma BDNF levels [8].

Vascular endothelial growth factor (VEGF) is a neurotrophic, neuroprotective, and neurogenetic factor, which is one of the most overexpressed angiogenic factors and encourages neurogenesis [8,9]. In addition, VEGF has a wide range of biological activities that include hematopoiesis, remodeling and survival of the vascular wall, vasculogenesis, vascular permeability, and inflammatory response modulation [10]. Animal studies with reduced VEGF levels showed amyotrophic lateral sclerosis (ALS) in humans, and increased VEGF expression delayed neurodegeneration and prolonged survival in ALS mice [11]. Another study also showed that physical exercise significantly downregulated VEGF gene expression in aging mice induced by D-galactose [12].

However, synthetic medications such as tacrine, donepezil, and galantamine, intended to treat learning and memory disorders, can halt the disease’s pathological progression but often come with unfavorable side effects [13]. In this regard, the experts conducted a lot of research to find and create medications that would treat cognitive disorders more effectively while causing fewer side effects.

Herbal plants containing antioxidants have been widely studied. Andaliman is also known as Bataknese pepper, an ethnic group in North Sumatra, widely consumed as an exotic spice in traditional cuisines and daily food. Hanum and Laila [14] demonstrated the antibacterial and anti-aging effects of Andaliman fruit ethanol extract (AEE) at a dose of 300 mg/ml for four weeks. In a different study, rats treated with 300 mg/kg bw of andaliman fruit ethyl acetate extract demonstrated cardioprotective effects [15]. However, it has never been documented that AEE slows down the oxidative stress-induced aging process. The aim of our investigation was to assess the impact of AEE on BDNF, VEGF, and hippocampus spatial memory in aging model rats.

## Materials and Methods

### Ethical approval

A control group that was only used for the post-test was used in the experimental investigation. Rats were given AEE and D-galactose as an aging model. The Universitas Sumatera Utara Medical Ethics Committee has approved the study’s protocol (No. 117/KEP/USU/2021). The “Guide for the Care and Use of Laboratory Animals” and the ARRIVE guidelines, which set forth animal ethics, were followed in this study.

### Collection and authentication of plants

Andaliman fruit used was dried andaliman fruit comes from the Samosir Dolok area, Ronggu Nihuta subdistrict, Samosir district, North Sumatra, Indonesia. This plant grows at an altitude of 1400 m above sea level. The andaliman fruits were harvested at a mature age, 6–8 weeks old. This plant has been determined and identified in the Herbarium Bogoriense, Biology Research Center-LIPI Bogor, including the type *Zanthoxylum acanthopodium* DC of the Rutaceae family.

### Preparation of andaliman fruit ethanol extract (Zanthoxylum acanthopodium DC)

The dried andaliman fruit was ground into a powder using a blender. Simplicia powder was macerated with 250 ml of 96% ethanol solvent for 24 h and then filtered to obtain filtrate and a dreg. The sediment was again macerated with 150 and 100 ml of ethanol for 24 h, then the macerated filtrate was combined and transferred to a 500 ml volumetric flask, then diluted with ethanol to the limit. The filtrate is allowed to stand for 24 h and then poured off. The filtrate is evaporated with a rotary evaporator at a temperature of 40°C–50°C and dried with frits (Indonesian Herbal Pharmacopoeia, 2017).

### Phytochemical screening

Phytochemical screening was performed using standard procedures based on the analysis of metabolites present in the ethanol extract of *Zanthoxylum acanthopodium* DC. Subsequently, the content of substances present in the ethanol extract of andaliman fruits was determined to be flavonoids, phenols, alkaloids, and antioxidants.

### Animals and treatments

Four groups of twenty-four male rats were created with simple random methods, namely N, M, P1, and P2. N (Normal): was given a standard diet; M (Model): aging model; 150 mg/kg bw of D-galactose was subcutaneously injected every day for eight weeks; P1: model + 150 mg/kg bw of AEE; and P2: model + 300 mg/kg bw of AEE. The rats were kept in stable conditions (temperature and humidity) in the animal cages and given stranded pellets and distilled water in compliance with laboratory protocol, according to the Biology Department of Universitas Sumatera Utara.

The inclusion criteria were Wistar rats (200–250 gm), male, and 12–14 weeks old. During the 7 days of observation before treatment, the rats were healthy and had normal activity and behavior. Exclusion criteria were dead rats or sick rats, inactive movement, and lost hair during the study process. The rats’ hippocampal organs were identified and preserved at 70°C after the rats’ cervical dislocation was used to dissect them. The examination of samples was carried out blindly; samples that had been labeled were closed and given new numbers randomly so that the examiners and researchers who participated in the examination did not know the sample being examined.

### Real time-PCR

Actin and glyceraldehide 3-phosphate dehydrogenase (GAPDH), two housekeeping genes, were measured by RT-PCR using particular primers for mRNA BDNF and VEGF (Elkins Park, PA, USA). Primer of BDNF: 5’-CAT TTC ATG ACA CTC GTG GA-3’ (forward) and 5’-ATT TCA GTG GCA GTG TGG AT-3’ (reverse). A primer for VEGF: 5’-GTG AGA AAA TGC TGG CCT AA-3’ (forward) and 5’-CTG CCA CAG GAA CTA GAG GA-3’ (reverse). Via the use of the RiboZol RNA extraction reagent kit (#N580, AMRESCO), total RNA was extracted from kidney tissue. Through the use of the iScript cDNA Synthesis Kit (#1708891, Bio-Rad Laboratory), complementary DNA (cDNA) was reverse transcribed. Using GoTaq^®^ Green Master Mix (Promega), the polymerase chain reaction (PCR) technique was used to amplify the isolation process. Two minutes at 95°C, 40 sec at 95°C for 5 sec, and 20 sec at 60°C were the RT-PCR steps that were employed. The C(q) value is computed automatically by Bio-Rad Laboratory’s CFX Manager TM v.3.1. The Rq, which is normalized to the control group or Rq = 2−ΔΔCt (ΔCt = Ct [target] – Ct [*β*-actin]; ΔΔCt = ΔCt [sample] – ΔCt [control]) was used to represent the mRNA expression [16].

### Test of the Morris Water Maze (MWM)

The MWM examination is used to assess memory, spatial learning, and cognition. The instrument consists of a circular water pool with a diameter of 200 cm, filled with water-mixed talcum powder as white as 66 cm, and is separated into four sections. In the middle of one of the quadrants, a little circular platform with a diameter of 65 cm is positioned and submerged 1 cm below the water’s surface. According to Zhang et al*.* [17], a training trial was conducted for five days in a row; each rat was trained four times a day to find a concealed platform within sixty seconds. Should the rat not locate it, it is artificially brought onto the platform, where it remains for fifteen seconds to retain its position.

To assess spatial learning abilities, the escape latency—the amount of time required to locate hidden platforms—was measured. In the probe test, the platform was taken out, and the rat’s frequency of crossing the platform’s location, as well as how long it spent in the target quadrant over the course of 60 sec, were noted and used to assess the rat’s spatial memory [17]. 

### Statistical analysis

The Livak formula was used to calculate each group’s relative expression of BDNF as well as VEGF. The effect of AEE on spatial memory and learning was analyzed with a one-way ANOVA test, but variables with an aberrant distribution (*p* < 0.05) were performed with a *Kruskal–Wallis* test.

## Results and Discussion 

### AEE activated the mRNA BDNF and VEGF in the hippocampus of an aged rat, which was induced by D-galactose

**Figure 1.**
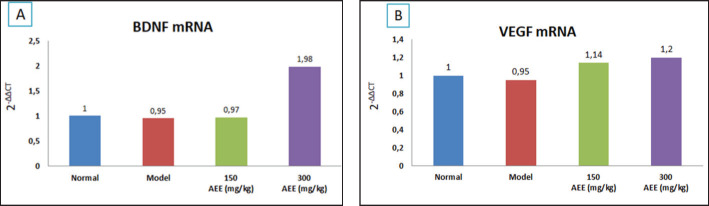
Andaliman fruit ethanol extract (AEE) activated mRNA expression of BDNF (A) and VEGF (B) of hippocampus in aging rat model D-galactose-induced.

Figure 1 illustrates how AEE at a dose of 300 mg/kg bw increased VEGF expression by 1.2 times and BDNF expression by 1.98 times. Model rat AEE administration was significantly up-regulated (*p* = 0.93 and *p* = 0.118) when compared to the normal and control groups.

AEE administration at a dose of 300 mg/kg had better results than the AEE at a dose of 150 mg/kg increase in BDNF expression compared to the normal group (1.98, 0.97 versus 1), listed in Figure 1A. This result indicated an increase in BDNF expression after high doses of AEE (300 mg/kg bw). AEE contains flavonoids, alkaloids, glycosides, tannins, and tritrerpenoids. The dried andaliman fruit used in this study came from the Samosir Dolok region, a district of Samosir. The total flavonoid contents of AEE were 21.0174 mgQE/gm via UV-Vis spectrophotometry, and the antioxidant level test showed an IC50 concentration of 108.11 ppm. Among the many dietary polyphenol compounds known as flavonoids, some have the capacity to trigger BDNF signaling [19]. Flavonoids inhibited neurologic disorders and ameliorated cognitive deficits in rodent models, potentially therapeutics in dementia. [20,21]. HAS of *Zanthoxylum bungeanum* Maxim (2.5 and 5.0 mg/kg) prevented alterations in the morphology and apoptosis of hippocampal neurons and increased the ACh content and mRNA expression of BDNF (*p* < 0.05) and p-CREB (*p* < 0.01) in contrast to those within the model group [18].

In this study, differences in VEGF expression between the eight-week treatment groups were observed. The results showed the expression of VEGF tended to rise in the treatment groups (doses of AEE: 150 mg and 300 mg) in contrast to the normal group (1.14; 1.2 versus 1), although there were no significant results (*p* = 0.118) as shown in Figure 1-B. There was evidence that suggested the flavonoids, particularly flavanols, were clinically capable of promoting cardiovascular improvements by lowering blood pressure, improving endothelial function, inhibiting platelet aggregation, and limiting inflammatory responses after oral administration [22,23]. Changes in peripheral vascular function facilitated efficient cerebral blood flow, which was worsened with age and decreased in patients with dementia. The flavonol contents in cocoa have also shown positive effects on blood flow to the brain via the artery of the middle cerebral cortex in humans [24]. Furthermore, Wang et al*.* [25] reported that flavanols increased cerebral blood flow no more than two hours after consumption. Improved blood vessel and cerebrovascular function is understood to promote mature neurogenesis in the hippocampal [25].

### AEE’s effects on spatial learning and memory in aging model rats induced by D-galactose

**Figure 2.**
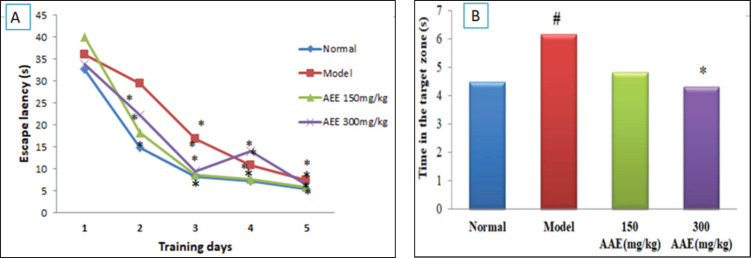
Effect of AEE on learning and memory deficits of D-galactose induced in Morris Water Maze (MWM) test. (A) The mean escape latency (5 days) to find the hidden platform in navigation training trials. (B). The time spent in the target quadrant. #*p* < 0.05 compared to normal control; *p < 0.05 compared to model group (D-galactose induced). Kruskal–Wallis test continued with post hoc Mann–Whitney.

To ascertain whether the rats were cognitively impaired, hippocampal-dependent spatial learning with additional memory behavioral tests using the Morris Water Maze was conducted [18]. As shown in Figure 2A, rats in the model group showed the longest escape latency (the time it took to reach the platform) between treatment groups, considerably longer than other rats with *p* = 0.001 (*p* < 0.05), via the *Kuskall–Wallis* test. These results indicated that model rats’ capacity for learning and memory was severely compromised. AEE therapy at a dose of 300 mg/kg can reduce the latency time in an aging model with significant results (*p* < 0.05).

The probe test showed significant differences between the group treatments (*p* = 0.026, (*p* < 0.05). A *post hoc* Mann–Whitney test was then conducted; travel time in the target zone of the normal group was shorter than that of the model group [4.5(4–5) versus 6.0(5–7), significantly *p* = 0.006. Interestingly, AEE administration at a dose of 300 mg/kg bw significantly reversed the adverse behavioral changes of aging models [4.5 (3–5) versus 6.0 (5–7)], *p* = 0.007 (Fig. 2B). AEE at a dose of 300 mg/kg bw significantly improved spatial learning and memory abilities.

Our findings also showed swimming time in the target zone between treatment groups with significant differences (*p* = 0.026) and, interestingly, AEE at a dose of 300 mg/kg bw (*p* < 0.05), as listed in Figure 2-B. A spatial learning and memory behavior of the hippocampus with an MWM test was conducted to ascertain whether the rats had cognitive impairments [18]. During the navigation training trials, researchers evaluated each rat for five consecutive days, conducting four evaluations per day. This study showed that the group of aging models displayed an extended escape latency to arrive at the platform during the exercise trial compared to a normal rat (*p* < 0.05), demonstrating that the aging model in rats had markedly reduced learning and memory. The results showed that giving AEE at a dose of 300 mg/kg bw significantly improved the capacity for spatial learning and memory in aged rats by enhancing the memory-related physiological response based on shortening the swimming test travel time of rats.

In this study, a daily dose of 300 mg/kg bw for eight weeks was able to reduce memory impairment, decrease the rate of aging, and prevent cognitive decline in rats given D-galactose to induce aging. This study’s limitation was that it only looked at BDNF expression in the hippocampal tissue. Depending on each compound’s degree of lipophilicity, flavonoids’ ability to cross the blood–brain barrier (BBB) must be taken into account. Less polar metabolites are more likely to do so than polar metabolites, and it is also necessary to consider the interaction of metabolites with specific transporters expressed in the BBB. Further research needs to be carried out regarding the level of brain bioavailability of flavonoids in humans.

## Conclusion

For eight weeks, andaliman fruit ethanol extract at a dose of 300 mg/kg bw had a tendency to raise hippocampal BDNF and VEGF expression, which markedly enhanced spatial memory.

## List of abbreviations

AEE; Andaliman fruit ethanol extract, BDNF; brain-derived neurotrophic factor, DNA; deoxyribonucleic acid, GAPDH; glyceraldehide 3-phosphate dehydrogenase, VEGF; vascular endothelial growth factor, ERK; extracellular signal-regulated kinases, PI3-K; phosphatidylinositol 3-kinase, PCR; polymerase Chain Reaction, Akt; protein kinase B, p-CREB; phospo-cAMP response element binding protein, BBB; blood–brain barrier.
